# Utilization of formal health services for children aged 1–5 in Aceh after the 2004 tsunami: Which children did not receive the health care they needed? Implications for other natural disaster relief efforts

**DOI:** 10.1080/21642850.2013.878658

**Published:** 2014-01-22

**Authors:** Bahie Mary Rassekh, Mathuram Santosham

**Affiliations:** ^a^ The World Bank, 1818 H St. NW, Washington, DC20433, USA; ^b^ Department of International Health, Johns Hopkins Bloomberg School of Public Health, 615 N. Wolfe Street, Baltimore, MD21205, USA

**Keywords:** rehabilitation, infants and toddlers, children and adolescents, immigrants or migrants

## Abstract

Aceh, Indonesia, was the hardest-hit area in the December 26, 2004 Indian Ocean earthquake and tsunami, with more than 500,000 people displaced, 120,000 people dead, and total damages and losses estimated at $4.5 billion. The relief effort following the tsunami was also immense, with billions of dollars of aid pledged to this province alone. Since then, there have been several natural disasters, including Typhoon Haiyan, which have caused great loss of life and displacement and for which these results are applicable. This study aimed to determine and assess utilization patterns of health services for children under the age of five with diarrhea, cough and difficulty breathing, fever, or skin disease and to identify determinants of formal and non-formal healthcare usage. A household survey of 1439 households was administered to caretakers of children aged 1–5 years. A sample of clusters within Banda Aceh and Aceh Besar were selected and those caretakers within the cluster who fit the inclusion criteria were interviewed. In the two weeks prior to the survey, 78.3% of respondents utilized formal health services as the first line of care for their child's illness episode. Factors significantly associated with decreased formal healthcare usage for the sick children were if the children were living in a displaced household, if the children's mother or father were not living, and if the children's caretaker was not the mother. Although utilization of formal health services for children was quite high after the tsunami, there were certain children who received significantly less care, including those who were displaced, those who were being cared for by someone other than their mother, and those for whom one or both parents had died. Among the recommendations are suggestions to target these children to ensure that they receive the health care they need.

True learning is that which is conducive to the well-being of the world … . (Bahá’í Writings)

## Introduction

On 26 December 2004, the Indian Ocean earthquake and massive tsunami caused one of the most devastating natural disasters in history, affecting hundreds of thousands of people. The hardest-hit country was Indonesia, and the province closest to the epicenter of the earthquake was Aceh, on the northern coast of Indonesia's Sumatra island.

**Figure 1.**
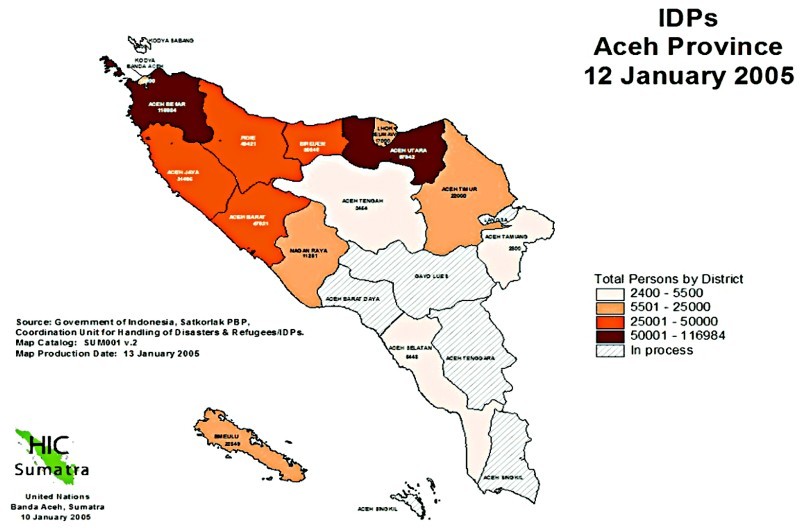
Internally displaced persons (IDPs) in Aceh province, January 2005 (Reliefweb, 2005).

In Aceh, the tsunami caused enormous devastation. More than 500,000 individuals were displaced there (Doocy et al., 2007), with the greatest density living in Banda Aceh and Aceh Besar in the north and Aceh Utara in the east (Figure 1). By a couple of months after the tsunami, around 400,000 people lived in camps in Aceh (Center for Communication Programs, 2005); 129,775 people were reported dead and 38,786 people were reported missing (Fitzpatrick, 2007), although in other data the death toll had reached approximately 200,000 and the number of internally displaced persons (IDPs) remained at around half a million (post-tsunami assistance risks neglecting reintegration needs of conflict-induced IDPs, 26 May 2005). The total population of the province was 4.8 million (WHO, 2005a), and so over 15% of the province's population was directly affected by the tsunami and was displaced, missing, or dead. By February, it was estimated that 21,659 houses and 1550 villages had been destroyed (WHO, 2005b). The International Organization for Migration reported that 66,000 houses were swept away in the tsunami, and 116,800 were damaged or destroyed (IOM, 2005). Of the 244 health facilities in the public sector, 53 (22%) were severely incapacitated or destroyed (WHO, 2005a) and of the province's 481 health professionals, 42 (9%) died (WHO, 2005a) and many others did not work after the tsunami. The total damages and losses were estimated at $4.45 billion, which is almost equal to the province's total GDP (BAPPENAS, 2005).

## International aid and relief work in Aceh

The scope and speed of the international aid and relief work was immense, nearly matching the magnitude of the damage and losses. Indonesia's Ministry of Health (MOH), agencies of the United Nations (UN), and over 250 non-governmental organizations contributed to this combined effort. The strategy was to strengthen coordination, share information on the epidemiological situation and disease surveillance, and do joint planning. By February, $4.3 billion of aid had been received in Indonesia and nearly half a million people received a one-month supply of food (United Nations News Service, 2005). The UN High Commission for Refugees had provided emergency assistance to 40,000 people within six weeks post-tsunami (United Nations News Service, 2005). Measures were taken to control mosquitoes after the tsunami, such as spraying insecticide in about 200,000 homes and providing bed nets and plastic sheeting treated with insecticide for homes in Banda Aceh (WHO, 2005c). At the peak of the relief effort during the first three months post-tsunami, medical supplies were being delivered. The World Health Organization (WHO) ensured supply of medical kits to cover the primary health needs of nearly two million people and provided surgical and diarrhea kits as well. In addition, more than 200 health experts were dispatched to work on tsunami-related health issues. Thousands of children were vaccinated against measles (WHO, 2005c).

Basic early warning and disease surveillance systems were put in place. Geographic information systems for constituting databases and health mapping for disease surveillance were also put in place. Local capacity was built by training provincial health office staff on how to enter and manage data regarding disease surveillance by WHO. Assessments of the water, sanitation, and health situations in IDP camps in Banda Aceh were conducted (WHO, 2005c). The MOH drew up an Action Plan for coordinating reconstruction and rehabilitation (Government of Indonesia, 2005).

By 20 months post-tsunami, the disaster reached the post-emergency rehabilitation and reconstruction phases. During the rehabilitation phase it was important to focus on two objectives: (1) evaluate program effectiveness by monitoring indicators and (2) determine the necessary actions and resources required for the transition from longer term relief to rehabilitation and reconstruction (Robinson, 2003). By then, IDPs had lived in camps, barracks, or other types of shelters for nearly 22 months, as the Indonesian government began providing barracks in which IDPs could reside approximately three months after the tsunami (personal communication between Dr Djoko and Dr Rassekh, September 19, 2005), and most IDPs who needed a place to live moved into barracks by August 2005.

The Indian Ocean tsunami brought with it enormous devastation, and an enormous global relief effort, with several billions of dollars pledged from organizations and governments across the globe. These events caused great changes in the lives of the Acehnese, especially those populations who were displaced from their homes and patterns of life. In order to fully support Aceh's reconstruction, health centers needed to be rebuilt and providers trained, but also more subtle behaviors of this vulnerable population had to be understood so that associated essential needs could be met.

## Understanding care-seeking behaviors

Much knowledge can be gained from understanding the practices of people affected by the tsunami in seeking health care for their children, applying some of these findings to other natural disasters, including Typhoon Haiyan. A successful health strategy depends only in part on the quality of a healthcare system. An equally important factor is the household's behavioral patterns, including care-seeking practices by a child's primary care-giver (Claeson & Waldman, 2000). A recommendation is that research strategies increasingly focus on better understanding care-seeking practices (Claeson & Waldman, 2000). There is abundant research that discusses the importance of understanding care-seeking practices in order to meet the health needs of disadvantaged populations (Aggarwal, Kumar, & Kumar, 2003; Barnes-Josiah, Myntti, & Augustin, 1998; Claeson & Waldman, 2000; Darmstadt et al., 2005; Fikree, Ali, Durocher, & Rahbar, 2004; Holts et al., 2003; Mbonye, 2003; Mecknonnen, 2003; Oluwole, Mason, & Costello, 2000; Preker & Carrin, 2004; Reyes et al., 1993; Walia & Kumar, 1984; World Bank, 1995). Understanding what enables people to utilize health care is important in order for health improvements to be made in populations where care-seeking is relatively infrequent. In the Lancet series on neonatal survival, six key knowledge gaps were identified, the first two of which related to knowledge about increasing care-seeking and the demand for quality care at the community level (Darmstadt et al., 2005). Care-seeking practices also include the type of care sought, since often care is sought from unproven informal sources. Two studies in India discuss this, demonstrating the high percentage of households that utilize home remedies or rural untrained medical practitioners (Aggarwal et al., 2003; Walia & Kumar, 1984). When as much as 80% or higher of total care costs can be from direct out-of-pocket payments in low-income countries, and the cost of formal care is high, some poor people prefer to use their scarce resources to seek care from informal practitioners, since the public health services are often inefficient, and drugs often must be additionally purchased after seeking care (Amarasiri de Silva, Wijekoon, Hormik, & Martines, 2001).

Studies show that there are several factors that influence care-seeking practices. One study found that significant factors associated with care utilization were child gender and child disease, household standard of living, and service availability and need (Thind, 2004). In this study, male children used more formal care than female children, and children with respiratory infections or with both this and diarrhea used more formal care than those with only diarrhea, adjusting for other variables (Thind, 2004). Other studies found that preference for traditional practices, poor knowledge for recognizing need for care, lack of transportation, high healthcare costs, and lack of confidence in the quality of care at the health facilities were barriers to care-seeking (Amarasiri de Silva et al., 2001; WHO Regional Committee, 2003). In terms of newborn health, poor care-seeking practices have been associated with type of illness, higher birth order, low paternal education, and lower household expenditure (Ahmed, Sobhan, Islam, & Barkat-e-Khuda, 2001). Other factors that are important include type of illness, lower economic status, lower maternal education, poorer type of residence, and lack of previous antenatal visit by the mother as important factors (Moss, Darmstadt, Marsh, Black, & Santosham, 2002; Pandey et al., 2002; Pillai et al., 2003). In a study in Uganda, even with a high rate of knowledge of childhood diseases, care-seeking was poor, suggesting that other barriers to accessing care exist, such as a limited traditional decision-making role of women (Mbonye, 2003). In a study in southern Iraq, the main determinants for utilization of care included level of perceived sickness and distance to the health facility (Habib & Vaughan, 1986). A World Bank report cited income, knowledge, gender of care-seeker, decision-making power of care-seeker, and community values and traditions as determinants of care-seeking (World Bank Consultation, 2002).

Data on care-seeking practices of displaced populations and populations in transition to resettlement show varied results. Under most conditions, care practices in disasters are very poor, and yet in some instances there is actual improvement in care-seeking (Madzokere, 1993; Miller, Jami-Imam, Tinouri, & Wijuker, 1995; Nabarro, 2005; Van Damme, De Bronwere, & Boelapart, 1998; WHO, 1999). In fact, one report states that leaps forward can be made through rehabilitation (Health Net International, 2001). Care-seeking behaviors are important to understand because a population's health status depends largely on healthcare usage (Pokhrel & Sauerborn, 2003). Much knowledge can be gained from understanding the practices of IDPs and others directly affected by the tsunami in seeking health care.

## Access to care

Access to care is composed of several elements, including distance to care from the home, access to transportation to care, and cost of transportation, health care, and drugs. These have been found to be important determinants of care-seeking in several studies (Ahmed et al., 2001; Buor, 2003; Frankenberg, 1995; Habib & Vaughan, 1986; Pandey et al., 2002; Pillai et al., 2003; Pokhrel & Sauerborn, 2003; WHO Regional Committee, 2003) and can likewise present great barriers to reaching care. It was found in a study in Ghana that greater distance to care was highly associated with poorer utilization, and that respondents were willing to travel up to 5 kilometers in order to reach care (Buor, 2003). In that population, among those people who lived within 30 minutes of care, 50% regularly used it, as opposed to around 3–4% of people who lived more than 30 minutes away from care. In a paper on access to health care in Indonesia, it was found that mortality rates were 40% higher for children who lived more than 10 miles from a hospital versus those who lived within 3 miles from a hospital, and 54% higher when a doctor is more than 5 miles away as opposed to in the child's village (Frankenberg, 1995). In this study, access to care was determined at the household level and compared to utilization of services for children who needed care in the two weeks prior to the survey. Additionally, IDP status and its association with access to care were considered.

## Focus on children under the age of five

There is a large and growing gap between rich and poor countries in terms of infant and child mortality (Claeson & Waldman, 2000; World Bank Health-Nutrition-Population, 2002). In poorer countries, children under the age of five bear 30% of the burden of disease (World Bank Health-Nutrition-Population, 2002). Approximately 70% of childhood deaths are due to preventable or treatable causes, such as ARIs, diarrhea, measles, malaria, and malnutrition (Claeson & Waldman, 2000). Health during childhood is especially important as early illness may lead to further illnesses later in life (Claeson & Waldman, 2000) and the health of a person throughout life is partially dependent on interventions early in one's life, or lack thereof (World Bank Consultation, 2002).

In order to better understand the process of managing illness in children leading from illness to health and survival, the Pathway to Survival is one model that can be considered, as it outlines the main elements involved in this process (Waldman, Bartlett, Campbell, & Steketee, 1996). Most quality care is provided for children with illness outside the home (Waldman et al., 1996), and therefore, the step that connects the mother and child to the health system, namely care-seeking, is critical. It is important to understand what leads to this step, and the percentages of caretakers who are choosing to take this step. In order to improve child survival, the determinants of healthcare utilization must be better understood. Then, relevant training can be provided and policies implemented to maximize healthcare usage (Thind, 2004). In Sri Lanka, a study found a correlation between high rates of seeking outside care for treatment and lower levels of childhood mortality (Amarasiri de Silva et al., 2001).

The caretaker must also recognize illness danger signs and respond in a timely way (Barnes-Josiah et al., 1998; Fikree et al., 2004; Holts et al., 2003). According to the ‘Three Delays Model’, which was originally used for maternal health care, there are three types of delays in obtaining care: (1) deciding to seek appropriate medical help, (2) reaching an appropriate health facility, and (3) receiving adequate care when a facility is reached (Barnes-Josiah et al., 1998). These barriers and determinants for care-seeking have been outlined in another way by Andersen as predisposing characteristics and enabling resources (Andersen, 1995). All of these models can be useful in understanding care-seeking behaviors and utilization of health services for caretakers of children under age 10 (Table 1).

**Table 1.** Determinants of care-seeking and associated interventions, the pathway to survival framework (Waldman et al., 1996).

Stage in framework	Determinants	Interventions
Caretaker seeks care outside the home	Knowledge:	Effective communication:
	(a) Recognition of need for additional care	(a) Content
	(b) Where to seek help	(b) Strategy
	(c) What is appropriate care	(c) Capabilities
	Access	Identification of options to increase access to appropriate care:
		(a) Organize existing services to facilitate access (hours, services available)
		(b) Expand existing services
		(c) Provide appropriate care through alternate channels (private/community; formal/informal)
	Decision to seek appropriate care (versus non-care or other care)	(a) Analysis of determinants of decisions regarding care-seeking (cost, perceived quality, cultural/social, previous experience)
		(b) Development/implementation/evaluation of interventions

## Caretakers of children in Aceh

In Aceh, the situation after the tsunami was quite unique. Thousands of mothers, the traditional primary caretaker of children under the age of five, had died. Half a million people were displaced. The care-seeking patterns of caretakers of children under the age of five in that population are extremely important to understand given the level of household movement and change, and shifts in household roles. Throughout this process the health needs of children under the age of five were potentially neglected. In order to enable child mortality levels to remain at a reasonable level throughout the resettlement process in other similar disasters, it is important to ensure that children who need health care are receiving it, and that caretakers of these children are seeking care appropriately.

Many children lost one parent in the tsunami and resided with the other parent and often other relatives. Understanding who cared for these IDP children and other household demographics was very important. Assessing associations between caretaker type and care-seeking behavior for sick children was essential in this analysis.

In this study, care-seeking practices of two populations were assessed: IDPs living in barracks and non-IDPs living in their permanent homes. Care-seeking practices of caretakers of children between the ages of one and five were considered. The conditions for care-seeking that were included were symptoms of the common illnesses that affected children under the age of five in this population, including diarrhea, cough and difficulty breathing, skin disease, and fever. The study also examined care-seeking practices for primary care-givers of these children by type of caretaker, including if the caretaker was the mother of the child or a non-mother, such as the father of the child, other family member, or a non-family member. The purpose was to determine if non-infant children under the age of five utilized the available formal healthcare resources when needed and to explore what determinants were associated with care-seeking in this population.

This historic event created a situation where living conditions, household structures, and household roles changed, and where trauma affected much of the population. With huge amounts of aid having been provided for Aceh, this evaluation of the situation in terms of children's access and usage of necessary primary care is critical. The research will contribute to a greater understanding of the effect of the tsunami and other similar disasters on childhood illness and survival in the long term, since care-seeking patterns of caretakers dictate childhood quality treatment receipt, which is necessary for child mortality to remain low in the years following such disasters.

This study was carried out in association with the Johns Hopkins University Center for Refugee and Disaster Response. It was part of the Center's evaluation of the health status and living conditions of IDPs in the Aceh region of Indonesia, affected by the tsunami.

## Methods and materials

### Study site

The population included in this study was already self-selected by IDP status, type of caretaker, and care-seeking behavior. Therefore, a quasi-experimental non-randomized design was utilized to fulfill the objectives of this study – to assess the care-seeking practices of two populations: IDPs living in barracks and non-IDPs living in their permanent homes.

A cross-sectional study design was used to study the relationship between care-seeking behavior at formal healthcare services and household IDP status for children under the age of 5.

The sampling method used was stratified cluster sampling, with sampling units of clusters of population within the Banda Aceh and Aceh Besar areas. Barracks existed in groups and were best divided as clusters of homes. Forty-eight clusters of 24 households each were to be interviewed in this study, since approximately 30 clusters enable study estimates to be precise (Robinson, 2003).

Disproportional stratification was used, such that the sample size for each strata was calculated independently of that characteristic's natural occurrence in the population. In this situation, it was important to utilize this method of stratified sampling in order to ensure that a detectable difference could be seen in the primary outcome measures.

In this study, inclusion depended on need for care as determined by the caretaker's recognition of the following conditions: Diarrhea, cough and difficulty breathing, skin disease, and fever. In this study, these symptoms were determined by the caretaker.

### Data collection

The main data collection instrument was a survey conducted by trained interviewers who were enrolled or recently graduated university students in Banda Aceh. There were four teams of six interviewers and one supervisor each. The survey was administered in the local language, Bahasa Indonesia, 9 to 10 months post-tsunami. The interviewers and supervisors were trained prior to the commencement of data collection and a field manual was given to each person to better understand the process and to refer to throughout the study.

In order to ensure that participants consented to being included in the study, interviewers read an introductory consent statement and asked all respondents to sign it before proceeding with the survey. In case the respondent was unable to sign it, verbal consent was given and the interviewer took note. In order to maintain confidentiality, participants' full names were not recorded on the survey. At the end of the interview, interviewers reviewed completed questionnaires in order to ensure that all questions were filled out completely and appropriately.

The survey was translated into Bahasa Indonesia from English, and then back-translated in order to ensure accuracy and maintain the integrity of the questions and their intended meaning. The survey was pilot-tested for approximately 30 individuals before official data collection commenced. This provided an opportunity to field-test the survey instrument and to further refine the questions in order to ensure accuracy and to enable evaluation of question precision in Bahasa Indonesia. Some questions were open-ended during the pilot test in order to allow caretakers to answer them as they saw fit. The data gathered from these questions were coded and common responses were added to the questionnaire for inclusion in the official data collection process. This also allowed an opportunity for project investigators to gain a better understanding of local customs and common practices used for treating childhood illness. This information enriched and strengthened the survey instrument and allowed for a more fruitful research process.

Although great care was taken to get accurate lists of all the barracks from various government offices, none of the lists accurately reflected all functioning barracks. Several barracks were listed that were not being used, mostly because people had not yet moved into them. This affected the study's household selection process. Originally, using the most complete list possible from the government office in Banda Aceh, barracks were randomly selected to be included in the study. Once teams went into the field to collect data, if a barrack was not in operation, then another barrack in the same area was chosen to be visited. If that barrack also was not operational, then the next barrack would be chosen, until one was found. Clustering in terms of non-IDP neighborhoods remained as it was intended to be. Overall, there were 31 clusters with 1295 observations.

Data were entered, and then re-entered, in Access by trained data-entry people locally hired in Indonesia. Double entry was conducted by a different person entering the same data for a second time. Discrepancies between the two sets of entered data were then checked against the original form. Double entry and discrepancy checking ensured accuracy and completeness of the data, so that it reflected the raw data as they were collected. Once data were entered, a thorough analysis was conducted.

The survey included questions regarding utilization of health services and characteristics of the caretaker, household, and child. These included the caretaker's age, education level, household income source and savings amount, number of children for whom the caretaker was caring, distance to the health facility from the home, age and gender of the child, and cost of services, among others.

### Setting and participants

The study was conducted in urban Banda Aceh, in the province of Aceh, Indonesia. This province was closest to the epicenter of the earthquake, was hit the hardest by the tsunami, and was home to over 500,000 displaced tsunami victims (Doocy et al., 2007) (Table 2).

**Table 2.** Selected overall health indicators in Indonesia, pre-tsunami (BPS Statistics Indonesia, 2006).

Selected indicators	1995	1997	1999	2001	2003
Percentage of population having health problems during the last month	25.38	24.41	24.70	25.49	24.41
Percentage of all infants immunized	77.34	91.01	89.91	n.a.	n.a.
Percentage of population who used traditional medicine	27.57	n.a.	15.04	30.24	30.67

Aceh's total land area covers 56,717 square kilometers (Aceh-Eye.Org, 2005). It is located at the northern part of the island of Sumatra and was greatly affected by the tsunami. Aceh had experienced political unrest for nearly three decades prior to the tsunami. In May 2003, over 40,000 troops were sent there to fight the Free Aceh Movement (GAM), which had an estimated 5000 rebels. In one sub-district, 265 of the 443 primary schools were burnt down (World Bank – SPADA Project Paper, 2005). In the province overall, 23.4% of villages and neighborhoods (jurisdictions in Aceh) reported conflict (Barron, Kaiser, & Pradham, 2004). Banda Aceh, the capital city of Aceh province, remained relatively clear of conflict throughout this time.

There are little data from Aceh before the tsunami due to the political situation. Most organizations were not allowed to work there, and the government had closed the province to nearly all foreigners. The Demographic and Health Survey has been completed several times in Indonesia, but the province of Aceh was excluded from this important source of information. Inferences of Aceh's indicators have been made based on country-level data (Table 3).

**Table 3.** Indonesia pre-tsunami country-level indicators (UNDP, 2006).

Indicator description	Year estimate	Indicator value
Total population (millions)	1975	134.4
	2002	217.1
Maternal mortality ratio, adjusted (per 100,000 live births)	2000	230
		
Infant mortality rate (per 1000 live births)	1970	104
	2002	33
Under-five mortality rate (per 1000 live births)	1970	172
	2002	45

In order to classify care-seeking behavior by type of care sought, Table 4 of types of care in Aceh was created.

**Table 4.** Health facilities in Aceh, Indonesia.

Formal care		Informal Care
Public providers	Private providers	Private providers
• Provincial hospital	• Private hospital	• Native healer
• Armed forces hospital	• Private physician	• Chinese healer
• Community health center (Puskesmas)	• Health provider practice (nurse or midwife)	• Traditional healer
• Community health center aids – health posts (Pustus)	• Mobile clinics (these primarily represent the relief effort in Aceh post-tsunami)	• Acupuncture
• Integrated services post (Posyandu)		• Reflection massage
		• Paranormal
		• Radiestesi (use pendulums)

Indonesia has a strong family planning program with a wide network of community health centers called ‘puskesmas’. Overall, the network had over 7100 health centers, 23,000 sub-centers, and 19,000 village maternity centers. There were an average of 18 paid staff, including 1.1 doctors, per health center. However, according to a World Bank report, random spot checks show that there is a 42% absenteeism rate for health workers at primary healthcare facilities (Lajouw, Pradham, Saadah, Sayed, & Sparrow, 2001). In post-tsunami Aceh, the Indonesian government decided to waive user fees for health services for one year (Women's Commission for Refugee Women and Children, 2005).

## Comparison groups and selection criteria

**Figure 2.**
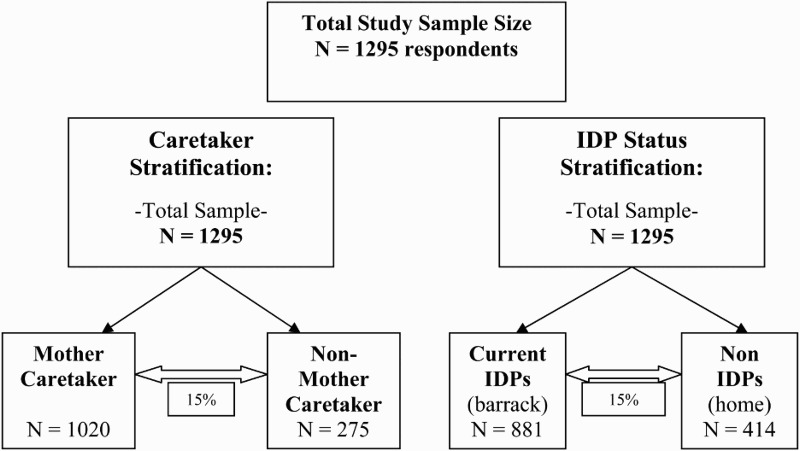
Survey sampling frame.

In this study, disproportional stratification was utilized, such that the sample size for each strata was calculated independently of that characteristic's natural occurrence in the population (Figure 2).

### Sampling frame for caretaker stratification

This stratification categorized participants by type of caretaker for the child under the age of five (mother of the child or non-mother). Therefore, of the 1295 households interviewed, there was about a 3:1 ratio of caretakers who were the child's mother to caretakers who were non-mothers. The sample size was calculated to allow for a power of 80% [(1−*β*) = 0.80] for a detectable difference of 15% in the outcome measure between these two populations.

### Sampling frame for IDP status stratification

This stratification categorized participants by IDP status. Therefore, two samples were utilized for this objective as well: 881 (68.0%) households of IDPs and 414 (32.0%) households of non-IDPs were included. This sample size was calculated to allow for a power of 80% [(1−*β*) = 0.80] to detect a difference of 15% in the outcome measure.

## IDP status comparison groups

### IDP households

#### Inclusion criteria

IDP who lived in a barrack community at the time of the survey and who had lived in a barrack community for at least two weeks were included in this category. Within the household there had to be a child between the ages of one and five who had a fever, cough and difficulty breathing, diarrhea, or skin disease in the two weeks prior to the survey.

### Non-IDP households

#### Inclusion criteria

People who lived in a home at the time of the survey, and who had lived in that home since before the tsunami, were included in this category. Within the household there had to be a child between the ages of one and five who had a fever, cough and difficulty breathing, diarrhea, or skin disease in the two weeks prior to the survey.

## Type of caretaker comparison groups

### Caretaker type: mother of child

#### Inclusion criteria

A child between the ages of one and five years who was cared for at the time of the interview by her/his mother was included in this category. That child had to be between the ages of one and five and have had fever, cough and difficulty breathing, diarrhea, or skin disease in the two weeks prior to the survey.

### Caretaker type: non-mother of child

#### Inclusion criteria

A child who was cared for at the time of the survey by anybody but her/his mother was included in this category. This included the child's father, other family member, or non-family member. That child had to be between the ages of one and five and have had a fever, cough and difficulty breathing, diarrhea, or skin disease in the two weeks prior to the survey.

### Results

The main results of this paper can be summarized as follows:

- 78.3% of respondents utilized formal health services as the first line of care for their child's illness episode in the two weeks prior to the survey.
- Factors significantly associated with decreased formal healthcare usage for a sick child were
  - if the child was living in an IDP household;
  - if the child's mother or father was not living;
  - if the child's caretaker was not the mother.

### Subjects

There were 1295 caretakers of children from 1295 different households who were interviewed in this study. Of these caretakers, 1014 (78.3%) utilized formal health services as their first line of health care for their sick child, and 281 (21.7%) did not use formal health care as their first line of health care. Non-formal health care includes traditional health care, home care, or no care for their sick child. Of these caretakers, 1253 were included in the multiple logistic regression analysis, representing 1253 unique households.

### Statistically significant factors

#### IDP status

Of the 1295 caretakers interviewed, 881 (68.0%) were IDPs and 414 (32.0%) were non-IDPs. Displacement was found to be an important variable in utilization of formal health services for children under the age of five by their caretakers. Non-IDPs were 2.4 times more likely to utilize formal health services than IDPs, in the crude analysis. A multiple logistic analysis was conducted, adjusting for variables of the child's age, type of caretaker, disease of child, if the child was the eldest, age and education of the caretaker, time to get to a formal healthcare facility, main reasons to return to health options again, living status of the child's parents, household savings, and whether the caretaker's mood was still affected by the tsunami. Using the adjusted multiple logistic regression (MLR), the odds of seeking formal health care for the sick child were also 2.7 times more for non-IDPs than their IDP counterparts. This was statistically significant at an alpha level of 0.05 (*p* = .004).

There was a slight difference between preference of care at the time of the study and before the tsunami, for IDPs versus non-IDPs. In general, IDPs reported greater preference, access, quality, usage, and lower cost for care than non-IDPs, after versus before the tsunami (Table 5). Overall, everyone, independent of IDP status, reported greater preference, access, quality, usage, and lower cost for care after the tsunami versus before the tsunami, but the difference was even greater for those who were displaced.

**Table 5.** Exploratory analysis: pre-/post-tsunami care preferences by IDP status (categorical variables).

	IDPs		Non-IDPs		Overall	*p*-Value
	*N*	%	*N*	%	*N* (%)	(chi-squared test)
*Prefer care*						
Before tsunami	135	23.04	55	27.09	190 (24.08)	.244
After tsunami (now)	451	76.96	148	72.91	599 (75.92)	
Total	586	100	203	100	789 (100)	
*Easier access to care*						
Before tsunami	114	17.17	52	24.30	166 (18.91)	.021
After tsunami	550	82.83	162	75.70	712 (81.09)	
Total	664	100	214	100	878 (100)	
*Better quality of care*						
Before tsunami	114	19.10	51	24.64	165 (20.52)	.089
After tsunami	483	80.90	156	75.36	639 (79.48)	
Total	597	100	207	100	804 (100)	
*Cheaper cost of care*						
Before tsunami	24	3.32	18	6.98	42 (4.28)	.013
After tsunami	699	96.68	240	93.02	939 (95.72)	
Total	723	100	258	100	981 (100)	
*More care used*						
Before tsunami	116	17.76	105	35.00	221 (23.19)	<.001
After tsunami	537	82.24	195	65.00	732 (76.81)	
Total	653	100	300	100	953 (100)	

#### Mother and father of child living

Of the 1295 households interviewed in this study, 1290 (99.6%) responded to this question. Of these 1290 households, 1073 (83.2%) had both the child's mother and father living, 86 (6.7%) had only the mother living, 94 (7.3%) had only the father living, and 37 (2.87%) had neither the child's mother nor father living at the time of the survey. Comparing each of the three latter categories of caretakers to those in which both the mother and father were living, each comparison produced highly significant results, and in each comparison there was a smaller likelihood of seeking formal care.

In this study, mothers and fathers of the sick child being alive was the single most important factor associated with seeking formal care. In the crude analysis (Table 6), formal care-seeking for a sick child occurred 85.01% of the time when both parents were alive, 51.00% of the time when only the mother was alive, 22.38% of the time when only the father was alive, and 11.36% of the time when neither parent was alive. When controlling for all other variables in the multiple logistic regression model, each of these latter three categories of children had significantly less usage of formal care than those children for whom both parents were alive.

**Table 6.** Exploratory analysis: usage of formal care by parent living status (categorical variables).

	Sought formal care		Did not seek formal care		Overall (*n* = 1425)	*p*-Value
	*N*	%	*N*	%	*N*	(chi-squared test)
*Mother and father of child living*						
Mother and father living	919	85.65	154	14.35	1073	<.001
Only mother living	49	56.98	37	43.02	86	
Only father living	31	32.98	63	67.02	94	
Neither mother nor father living	10	27.03	27	72.97	37	
Total	1009	78.22	281	21.78	1290	

**Figure 3.**
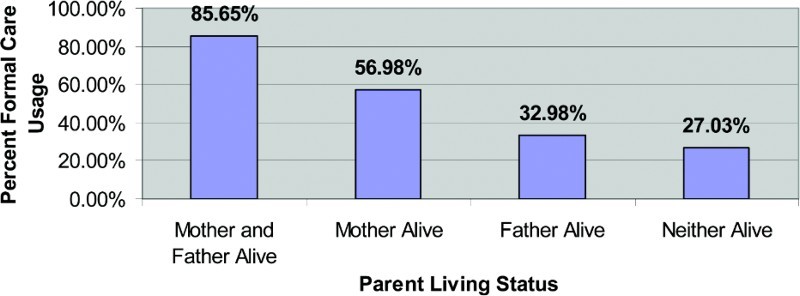
Usage of formal health care by parent living status.

Even after adjusting for all other variables in the multiple logistic regression, including type of caretaker for the child, this variable remained statistically significant (*p* < .001) for all categories (Figure 3).

#### Caretaker of child

Of the 1295 households interviewed in this study, 1295 (100%) responses were provided. Of the respondents, 1020 (78.8%) of the primary caretakers of the child were the child's mother, and 275 (21.2%) were non-mother caretakers, including the father, other family members, or non-family members. Whether the caretaker of the sick child was the child's mother or not was found to be a significant factor associated with formal care usage. Mother caretakers were 4.9 times more likely to take sick children to formal care in the simple logistic regression (*p* < .001). After adjusting for other variables in the multiple logistic regression model, mother caretakers had 1.9 times the odds of taking their sick child to formal care than their non-mother caretaker counterparts. This difference was borderline significant (*p* = .036).

### Borderline significant factors

**Figure 4.**
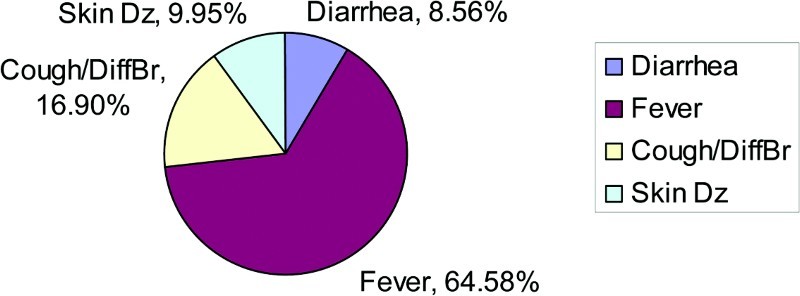
Disease prevalence for children aged 1–5 who had diarrhea, skin disease, cough and difficulty breathing, or fever – single illness.

**Figure 5.**
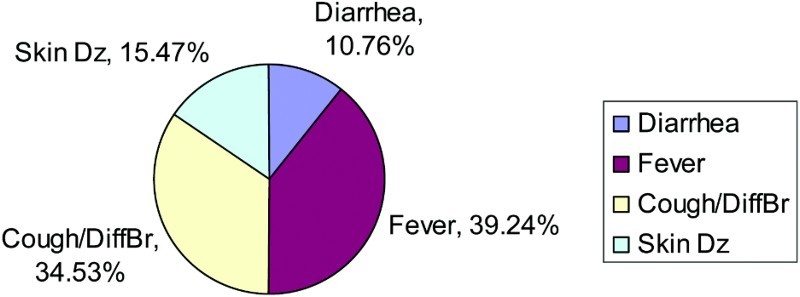
Disease prevalence for children aged 1–5 who had diarrhea, skin disease, cough and difficulty breathing, or fever – multiple illnesses.

There were no variables in this analysis that were borderline significant, with *p*-values between .050 and .100 (Figures 4 and 5).

### Non-significant factors

Several variables in the multiple logistic regression were not significantly associated with formal health services utilization. These variables included age of the child, gender of the child, disease of the child, whether the child was the eldest child, caretaker's age, caretaker's education, distance to care, caretaker's main reasons for returning to a healthcare option, household savings, and whether the tsunami still affects the mood of the caretaker (Table 7).

**Table 7.** Crude and adjusted odds ratios of utilization of formal versus non-formal health care.

	Crude (*n* = 1295)			Adjusted (*n* = 1253)		
	Odds ratio	95% CI	*p*-Value	Odds ratio	95% CI	*p*-Value
*IDP status*	2.445	(1.763, 3.392)	.000	2.738	(1.377, 5.444)	.004
*Age of child* (years)	0.915	(0.808, 1.036)	.161	1.032	(0.917, 1.160)	.605
*Gender of child* (female versus male)	0.909	(0.697, 1.186)	.483	0.812	(0.586, 1.126)	.212
*Caretaker type*						
Mother versus non-mother caretaker	4.934	(3.682, 6.612)	.000	1.881	(1.041, 3.396)	.036
*Mother and father of child living*						
Reference = both mother and father living						
Only mother living	0.222	(0.140, 0.351)	.000	0.251	(0.140, 0.450)	.000
Only father living	0.082	(0.052, 0.131)	.000	0.172	(0.082, 0.365)	.000
Neither living	0.062	(0.029, 0.131)	.000	0.142	(0.069, 0.290)	.000
*Child's illness*						
Reference = no diarrhea and no cough and difficulty breathing (cough/diff br)						
Diarrhea but no cough/diff br	2.223	(1.222, 4.062)	.009	1.386	(0.708, 2.710)	.341
Cough/diff br but no diarrhea	1.691	(1.261, 2.268)	.000	1.273	(0.853, 1.902)	.237
Diarrhea and cough/diff br	1.213	(0.786, 1.871)	.384	1.048	(0.544, 2.019)	.888
*First/eldest child* (yes versus no)	0.703	(0.537, 0.920)	.010	0.822	(0.599, 1.129)	.226
*Age of caretaker* (years)	0.967	(0.952, 0.982)	.000	1.005	(0.981, 1.030)	.677
*Education of caretaker* (years school attended) (>12 years versus <12 years)	1.048	(0.804, 1.365)	.730	0.857	(0.574, 1.281)	.452
*Household Assets*						
Reference = 0 assets						
1 asset	0.961	(0.697, 1.326)	.811	0.867	(0.568, 1.325)	.510
2 assets	1.049	(0.705, 1.562)	.812	0.834	(0.507, 1.371)	.474
3 assets	1.343	(0.773, 2.334)	.296	1.022	(0.579, 1.806)	.940
4 assets	1.372	(0.734, 2.567)	.322	0.624	(0.191, 2.041)	.435
*Distance to health facility* (Meters, 0–15,200 m)	1.000	(1.000, 1.000)	.962	1.000	(1.000, 1.000)	.888
Spline term (more than 15,200 m)	1.000	(1.000, 1.000)	.657	1.000	(1.000, 1.000)	.565
*Main reason return to health option again*						
Reference = good cost						
Easy to access	1.219	(0.874, 1.701)	.244	1.182	(0.723, 1.934)	.505
Good quality care/provider	0.959	(0.693, 1.328)	.801	1.065	(0.678, 1.671)	.786
Family supports it	0.620	(0.210, 1.829)	.387	0.495	(0.094, 2.592)	.405
*Tsunami affects mood now* (no versus yes)	0.000	(0.000, 0.000)	.000	0.921	(0.639, 1.328)	.661

### Effect modification

Effect modification was tested for several variables including: if the child was the eldest and child's age, child's gender, and parent living status; IDP status and caretaker type, child illness, distance to care, main reason the to return to a care option, parent living status, and if the caretaker's mood is affected by the tsunami; parent living status and child gender, caretaker type, child illness, caretaker age, caretaker education, reason return to care option, and if the caretaker's mood is affected by the tsunami. None of these were all statistically significant in a model that converged (Table 8).

**Table 8.** Exploratory analysis: characteristics of the formal care provided according to IDP status (continuous variables).

	IDPs (*n* = 651–755)		Non-IDPs (*n* = 349–409)		Overall (*n* = 1000–1164)	
	Mean	SD	Mean	SD	Mean	*p*-Value (*f*-test)
Distance between home and formal facility used (m)	1102.8	2388.1	1549.9	2191.3	1259.9	<.002
Time to reach formal facility used from home (minutes)	13.0	22.5	15.3	27.4	13.8	.157
Time waited before seeing health care provider (minutes)	14.8	19.9	18.2	23.5	16.0	.015
Cost of services (rupiah)	2805.4	22,171.6	7041.6	21,173.4	4261.6	.002
Cost of medicine (rupiah)	4088.1	18,321.4	14,000.0	32,645.9	7592.3	<.001
*Delays in care-seeking*						
Average number of days after first seeing symptoms that caretaker went to formal care option	1.23	1.19	1.42	1.79	1.30	.030
*Number of care options sought during this illness episode*						
Average number of healthcare options sought for child's illness episode total (not including ‘0’)	1.76	3.7% sought no care	1.70	22.0% sought no care	1.74	<.001 (including 0 values)

## Discussion

In summary, this paper puts forth that, although utilization of formal health services for children was relatively high after the tsunami, there were certain children who received significantly less care, including those who were displaced, those who were being cared for by someone other than their mother, and those for whom one or both parents had died. As part of the discussion that follows, it is suggested to target these children in particular to ensure that they receive the health care they need in the event of future natural disasters regardless of type or location.

It is emphasized by WHO that conducting an immediate post-disaster needs assessment is crucial for relief work, but that data should continue to be collected for several years after disasters (Nabarro, 2005). This is important in order to understand the behaviors and changing needs of the resettling populations, so that assistance can be planned and managed more effectively (Nabarro, 2005). Household care-seeking and utilization practices is one such form of data.

In the study setting, the average distance between the home and formal facility used was 1.2 kilometers, and it took an average of about 12.5 minutes to travel between the home and facility. The cost of services was 4264 rupiah on average (0.43 USD); although for a large percentage of the users, services were actually free, since the government provided free health care as did many relief organizations. Overall, of the 1295 respondents, 1014 (78.3%) sought formal care as their first line of care. Of the 281 people who did not seek formal care as their first line of care, 134 (47.9%) eventually sought formal care for this illness episode. Overall, 88.6% of all caretakers reported seeking formal health care for their sick child eventually during the illness episode for which the interview was conducted. This represents high utilization for childhood illness. In a study in Sri Lanka, a reason given to explain the low level of childhood mortality was a high percentage (65.0%) of care-seeking for childhood illnesses. This included traditional and other types of health care (Amarasiri de Silva et al., 2001). From the study results in Aceh, 78.3% of caretakers sought formal care as the first line of healthcare and 88.6% sought it during the illness episode was quite high for such a setting.

In this study, the most important determinants of healthcare utilization were such household characteristics as displacement, the number of people living in the household, and whether the child's parents were alive. These are not factors that can be easily prevented in most disaster settings. They are effects of the disaster that have been associated in this study with whether children receive formal care when they are ill. The primary determinant of utilizing health care for a child when s/he was ill was whether the mother and father were both alive, controlling for all the other variables in the multiple logistic regression analysis. This factor affected 16.8% of children from the study, which is a large proportion. Of the children asked about in the study, 6.7% had only their mother living, 7.3% had only their father living, and 2.9% had neither their father nor mother living.

Those who were not displaced had 2.7 times the odds of seeking formal care as their first line of care sought than those who were displaced adjusting for all other variables in the MLR. Of those respondents who never sought formal care for their sick child, 88.9% of IDPs and 54.6% of non-IDPs knew of at least one formal care facility that was reachable that could be used for their child. This difference between IDPs and non-IDPs was significant at an alpha level of 0.05 (*p* = .001). Therefore, of those caretakers who did not seek formal care, those who were displaced had greater odds of knowing of at least one accessible formal health facility for their child than those who were not displaced. This is particularly interesting given that formal health care was closer, required less waiting time, and was reported as less costly for those who were displaced compared to those who were not displaced. In many studies, it has been shown that these access-related factors are important determinants in healthcare utilization (Ahmed et al., 2001; Buor, 2003; Frankenberg, 1995; Habib & Vaughan, 1986; Pandey et al., 2002; Pillai et al., 2003; Pokhrel & Sauerborn, 2003; WHO Regional Committee, 2003). Despite having better access to health care, IDPs tended to use less care than their non-IDP counterparts. This can potentially be attributed to a lower relative priority for children's health for those who were displaced. A reason could be that these populations may have had other pressing priorities, as their environments were new and they were residing in temporary homes. Priority may have been given to such things as re-establishing their home, jobs, and other basic elements of their lives. In order to ensure that children receive health care when needed, more outreach efforts, such as home visits, may be required for those who are displaced.

Post-traumatic stress may also have affected the daily activities of displaced populations, including care-seeking behaviors. Trauma can occur in people due to personally experiencing a traumatic event, or witnessing trauma such as the death of another person (Jong, Mulher, & Can der Kam, 2000; Redwood-Campbell & Riddez, 2006), and the distress may be present even years later (Thomas, 2004). According to the ‘Three Delays Model’ which was originally used in the setting of maternal health care, the first of the three types of delays in obtaining care is deciding to seek appropriate medical help (Barnes-Josiah et al., 1998). This step relies on the initiative of the care-giver. In post-tsunami Sri Lanka, posttraumatic stress disorder's (PTSD) prevalence ranged from 14% to 39% (Neuner, Schauer, Catani, Ruf, & Elbert, 2006). Caretakers of children may have sought less health care for their sick child when suffering from PTSD, which may have been more prevalent among displaced populations.

In addition to displacement status, whether the child's mother or father was alive was a highly significant factor associated with care usage. It was in fact the most significantly associated variable in the MLR. This finding demonstrates the importance of a child's parents being alive for proper care-seeking to take place, independent of who is caring for the child, the caretaker's education level or age, and the other factors that were adjusted for. The most significant difference in care-seeking was seen when the mother was not alive. Therefore, interventions must focus on children whose mother or both parents are not alive, even if they are being cared for by another family member or other person.

Those caretakers with some savings used less formal care than their counterparts with no savings. This may be due to the pride associated with seeking free health care, as most public formal care was offered without cost during the time of the survey in Aceh. An example of this was illustrated in Sri Lanka, where focus group discussions carried out under the auspices of UNDP found that a stigma may have been attached to those affected by the tsunami and that some people felt embarrassed to depend on the relief provided (Disaster Relief Monitoring Unit of the Human Rights Commission of Sri Lanka, 2005). This could have been a hindrance for some displaced people to seek care and may have contributed to the study findings.

Interventions in other post-disaster settings, such as that associated with Typhoon Haiyan, could take findings from this study into consideration. For example, interventions could focus on IDP children, as their caretakers tended to seek care less than their non-IDP counterparts, despite having relatively better access to formal health services. They could also focus on children for whom one or both parents had died, as their caretakers tended to use much less care than their counterparts with living parents. In order to increase utilization of formal health services in similar settings in the future, factors shown in this study to be significant determinants of care-seeking could be considered. Those caretakers who have lower odds of seeking health care should be targeted with some form of intervention.

In a paper by Winch et al. (2005), various models of community health worker interventions were summarized. Due to the high incidence of acute respiratory infections in Aceh after the tsunami and low incidence of malaria (Eurosurveillance, 2005) intervention Model 6 or 7 from the paper may be best suited to this setting. Intervention Model 6 is called ‘Community health worker (CHW) pneumonia case management’ and CHWs assess and treat with antibiotics children who have signs of pneumonia. In this intervention, CHWs visit households monthly (Winch et al., 2005). After another similar disaster, it may be necessary for CHWs to visit households more frequently, such as every week after the disaster for the duration of the emergency phase, followed by bi-weekly visits to households of priority children. Priority children are defined as those who are displaced. High priority children are those who have lost their mother or both parents, even if they are being cared for by another family member.

An intervention like this may aid in increasing usage of the health facilities. The following cases present other examples of successful relief-related interventions. A study in Mozambique found that care-seeking practices can change and that the future of women is likely to change due to skills learned in refugee camps (Madzokere, 1993). A Knowledge, attitude, and practice surveys of Afghan refugee women shows that through relief work birth attendants could be trained, producing significant results in the care-seeking behavior of women and in the improved health of mothers and their newborn babies (Miller et al., 1995). In Guinea, it was found that rates for major obstetric interventions increased due to the availability of better services from pre- versus post-refugee data (Van Damme et al., 1998; WHO, 1999). In Afghanistan, another study demonstrated that use of health facilities increased significantly following relief work which included the training of female staff and better-equipped facilities with greater drug availability (Health Net International, 2001). In Cambodia, as a result of an intervention by relief workers, patient satisfaction increased, referrals between the traditional sector and the public health system increased by 150% in two years, and utilization of reproductive healthcare services increased by 20% (Health Net International, 2001). Finally, in East Timor, 77% of health facilities were destroyed due to conflict, and yet following relief work there, there was a greater equity of access to care, for both routine and emergency care (Health Net International, 2001).

After the Typhoon Haiyan and other similar disasters, interventions can focus on ensuring children have access to health care for those children whose mother or father have died and for those who are displaced. It may be beneficial to have health workers visit children's homes soon after the disaster to ensure that they are receiving care when needed. If the disaster causes displacement, then these displaced populations should be targeted. If the disaster causes a lot of death, especially in parents of young children, these children should be targeted.
